# Effects of Proton Pump Inhibitors and H_2_ Receptor Antagonists on the Ileum Motility

**DOI:** 10.1155/2011/218342

**Published:** 2011-12-14

**Authors:** Atilla Kurt, Ahmet Altun, İhsan Bağcivan, Ayhan Koyuncu, Omer Topcu, Cengiz Aydın, Tijen Kaya

**Affiliations:** ^1^Department of General Surgery, Faculty of Medicine, Cumhuriyet University, 58140 Sivas, Turkey; ^2^Department of Pharmacology, Faculty of Medicine, Cumhuriyet University, 58140 Sivas, Turkey

## Abstract

*Objectives*. To investigate the effects of proton pump inhibitors (PPIs) and H_2_ receptor antagonists on ileum motility in rats with peritonitis and compare changes with control group rats. *Methods*. Peritonitis was induced by cecal ligation and puncture in 8 rats. Another of 8 rats underwent a sham operation and were accepted as controls. Twenty-four hours later after the operation, the rats were killed, and their ileum smooth muscle was excised and placed in circular muscle direction in a 10 mL organ bath. Changes in amplitude and frequency of contractions were analyzed before and after PPIs and H_2_ receptor blockers. *Results*. PPI agents decreased the motility in a dose-dependent manner in ileum in both control and intraabdominal sepsis groups. While famotidine had no significant effect on ileum motility, ranitidine and nizatidine enhanced motility in ileum in both control and intraabdominal sepsis groups. This excitatory effect of H_2_ receptor antagonists and inhibitor effects of PPIs were significantly high in control group when compared to the peritonitis group. The inhibitor effect of pantoprazole on ileum motility was significantly higher than the other two PPI agents. *Conclusions*. It was concluded that H_2_ receptor antagonists may be more effective than PPIs for recovering the bowel motility in the intraabdominal sepsis situation.

## 1. Introduction

Sepsis is a systemic response to infection and inflammation. Sepsis syndrome which developing in reaction to sepsis has constituted one of most important causes of the mortality at present, despite, widespread use of specific antibiotics and other pharmacological agents [1–3].

 Sepsis is a syndrome that affects the public health system and represents a challenge to health care providers and managers. Epidemiological data revealed a high incidence of sepsis in patients hospitalized in intensive care units (ICU) compared with the occurrence of disease in general population [4, 5].

Dysmotility of the gastrointestinal tract is a major complication in critically ill patients in intensive care units (ICU). Most of the time, this dysmotility manifests itself as inhibition of gastrointestinal motility, and rarely as hypermotility [6]. Impaired motility in critically ill patients can be caused by intestinal ischemia, electrolyte imbalances, peritoneal injury, abdominal surgery, lower-lobe pneumonia, pancreatitis, cholecystitis, intraabdominal abscesses, and medications (opiates, dopamine, diltiazem, verapamil, and anticholinergics) [7].

Many critically ill patients in ICU require acid suppressive agents. Proton pump inhibitors (PPIs) are commonly used in the (ICU) for stress ulcer prophylaxis therapy given their potent acid inhibitory effect, an efficacy that is at least as good as, and perhaps better than, that of H_2_ receptor blockers therapy, and a benign safety profile [8, 9]. Although it is well known that peritonitis affects gastrointestinal system motility, patients suffering from peritonitis are good candidates for ICUs; however, PPIs and H_2_ receptor blockers are commonly used agents in these units; there is very little information about the effects of acid suppressive agents (ASD) on the intestinal motility in the patients. In this study, we aimed to investigate the effects of PPIs and H_2_ receptor blockers on ileum motility in both normal and peritonitis situations.

## 2. Materials and Methods

### 2.1. Animal Preparation

Sixteen male Wistar albino rats each weighing approximately 280 g were used in this study. The study was approved by the ethics committee of the Cumhuriyet University School of Medicine. Cecal ligation and puncture were used as the peritonitis model [10]. Animals were divided into two groups. The first group (*n* = 8) consisted of sham surgical controls that underwent the same procedure as the peritonitis group, such that laparotomy was performed under anesthesia, with manipulation of the cecum, but cecum ligation and puncture were not performed. Rats in the second group (*n* = 8) underwent cecal puncture and ligation as previously described by Richardson et al. [11]. Animals were anesthetized with intramuscular injections of 3 mg/kg xylazine (Rompun, Bayer, Istanbul, Turkey), and 90 mg/kg ketamine (Ketalar, Pfizer, Istanbul, Turkey), following which, laparotomy was performed via a 2 cm midline incision and the cecum was exposed. The cecum was ligated using 4/0 silk suture material just below the ileocecal valve, so that intestinal continuity was maintained. Then, the cecum was punctured using an 18-gauge needle in three locations, 1 cm apart, on the antimesenteric surface of the cecum, and cecum was gently compressed until feces were extruded. The cecum was replaced into the peritoneal cavity, and the abdomen was then closed. A summary of the experimental treatments is presented below, Groups: Group I (*n* = 8): sham surgical controls; Group II (*n* = 8): peritonitis group. At the second laparotomy, 24 h later, the rats were killed by cervical dislocation. The abdomen was opened with a midline incision, and the ileum was removed and placed in previously aerated (95% O_2_ and 5% CO_2_) Krebs bicarbonate solution (composition in mmol/L: NaCl, 120; KCl, 4.6; CaCl_2_, 2.5; MgCl_2_, 1.2; NaHCO_3_, 22; NaH_2_PO_4_, and glucose 11.5). Whole full-thickness segments of ileum were placed in circular direction in a 10 mL tissue baths, filled with preaerated Krebs bicarbonate solution (KBS) at 37°C. The upper end of the preparation was tied to an isometric transducer (Grass FT 03, Quincy, Mass, USA) and preloaded with 1–1.5 g. Tissues were allowed to equilibrate for 30 min.

### 2.2. *In Vitro* Muscle Contractility Studies

Muscle segments from each group were contracted with 80 mmol/L KCl to ensure that they worked properly at the beginning and end of each experiment. At the beginning of each experiment, 80 mmol/L KCl was added to the organ bath, and the contraction was considered as reference response. Subsequently, the amplitude of spontaneous contractions of the isolated ileum muscle segments was calculated as a percentage of the contraction induced by KCl (80 mmol/L) from both control and peritonitis groups. Changes in the frequency (number/min) of spontaneous contractions were expressed as the number of contractions for 10 min intervals. Following the KCl response, smooth muscle segments were allowed to equilibrate for 30 min before addition of cumulative doses of omeprazole (10^−8^–10^−4 ^mol/L), pantoprazole (10^−8^–10^−4 ^mol/L), lansoprazole (10^−8^–10^−4 ^mol/L), and famotidine (10^−8^–10^−4 ^mol/L), ranitidine (10^−8^–10^−4 ^mol/L), and nizatidine (10^−8^–10^−4 ^mol/L). The changes of amplitudes of the contractions induced by these compounds from both control and peritonitis groups were calculated as the percentage of the initial spontaneous contractions. Changes in the frequency of spontaneous contractions were expressed as the number of spontaneous contractions for 10 min after drug application. Isometric tensions were recorded on a Grass model 79 E polygraph.

### 2.3. Drugs

The following compounds were used: omeprazole, pantoprazole, lansoprazole, and famotidine, ranitidine, nizatidine (Aldrich Chemicals Co., USA). All drugs were dissolved in distilled water. All drugs were freshly prepared on the day of the experiment.

### 2.4. Data Analysis

All data are expressed as mean ± SEM. Statistical comparisons between groups were performed using general linear models of analysis of variance (ANOVA) followed by the Tukey test and a *t*-test when appropriate, and *P*  values of less than 0.05 were considered to be statistically significant.

## 3. Results

**Figure 1:**
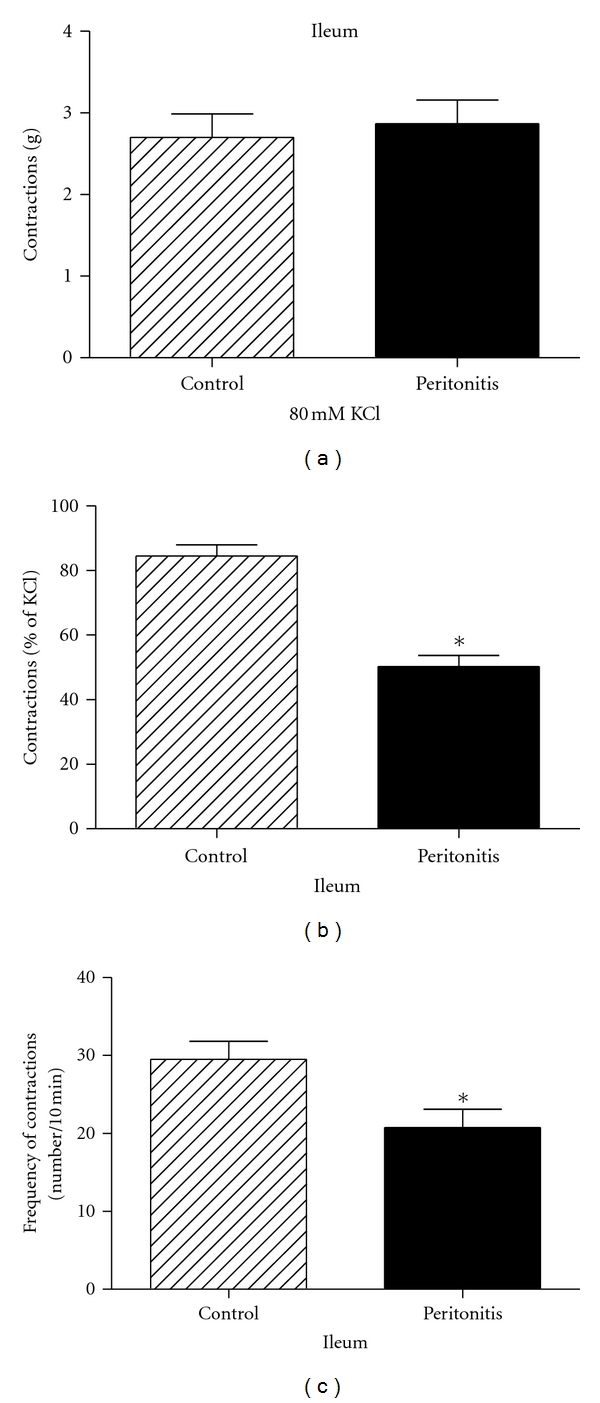
(a) KCl (80 mmol/L) induced contractions of isolated ileum muscle segments in control and peritonitis groups. (b) Changes in the amplitude of spontaneous contractions of the isolated ileum muscle segments. Amplitudes were calculated as a percentage of the contraction induced by KCl (80 mmol/L) from both control and peritonitis groups. (c) Changes in the frequency of spontaneous contractions of the isolated ileum muscle segments. Frequencies were expressed as the number of contractions for 10 min from both control and peritonitis groups. (*P < 0.05 versus control group; analysis of variance followed by Tukey test.)

Contractions induced by 80 mmol/L KCl were not significantly different between the peritonitis group and the control group in isolated ileum smooth muscle segments which indicated that muscle segments from both groups worked properly (Figure 1(a)).

 The mean amplitude of the spontaneous contractions was % 84.5 ± 3.4 of KCl in the control and % 50.2 ± 6.5 of KCl in the peritonitis group, respectively. The number of spontaneous contractions obtained in 10 min in the control group was 31.7 ± 2.6 and 20.8 ± 1.9 in the peritonitis group. Both the amplitude and the frequency of spontaneous contractions of ileum smooth muscle segments were significantly low in the peritonitis group when compared to the control group (*P* < 0.05, Figures 1(b) and 1(c)).

**Figure 2:**
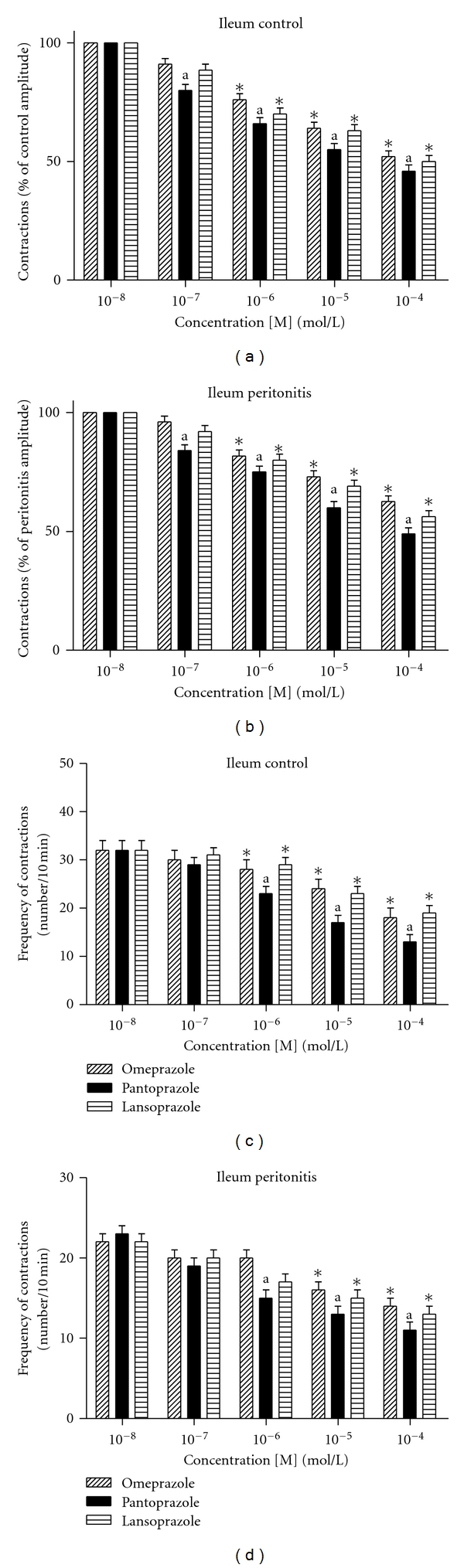
Amplitudes of the contractions induced by omeprazole, pantoprazole, and lansoprazole. (a) Control group; (b) peritonitis group; both were calculated as the percentage of the initial contractions. (*P < 0.05 versus initial contractions,  ^a^ *P* < 0.05 versus omeprazole and lansoprazole; analysis of variance followed by Tukey test.) Changes induced by omeprazole, pantoprazole, and lansoprazole in the frequency of spontaneous contractions. (c) Control group; (d) peritonitis group. Both were expressed as the number of contractions for 10 min. (*P < 0.05 versus initial contractions,  ^a^ *P* < 0.05 versus omeprazole and lansoprazole; analysis of variance followed by Tukey test.)

The amplitudes of spontaneous contractions of ileum muscle segments were studied after adding omeprazole, pantoprazole, and lansoprazole to the organ bath. Omeprazole (10^−8^–10^−4 ^mol/L), pantoprazole (10^−8^–10^−4 ^mol/L), and lansoprazole (10^−8^–10^−4 ^mol/L), significantly decreased the amplitude of spontaneous contractions, starting from 10^−6 ^mol/L for omeprazole and lansoprazole, in control group. However, this decreasing effect started at the concentration of 10^−5 ^mol/L in peritonitis group. In both groups, the inhibitor effect of pantoprazole on ileum motility was significantly higher than omeprazole and lansoprazole (Figures 2(a) and 2(b); (Table 1) (*P* < 0.05).

The frequency of spontaneous contractions of ileum muscle segments was studied after adding omeprazole, pantoprazole, and lansoprazole to the organ bath. Omeprazole (10^−8^–10^−4 ^mol/L), pantoprazole (10^−8^–10^−4 ^mol/L), and lansoprazole (10^−8^–10^−4 ^mol/L) was significantly decreased the frequency of spontaneous contractions starting from 10^−5 ^mol/L for omeprazole and lansoprazole, in isolated ileum muscle segments, in both control and peritonitis groups (*P* < 0.05). In both groups, the inhibitor effect of pantoprazole on ileum frequency, which was starting from 10^−6 ^mol/L, was significantly higher than omeprazole and lansoparazole. The inhibitor effect of PPIs on frequency of ileum smooth muscles was higher in control group when compared to peritonitis group (Figures 2(c) and 2(d); (Table 1) (*P* < 0.05).

**Figure 3:**
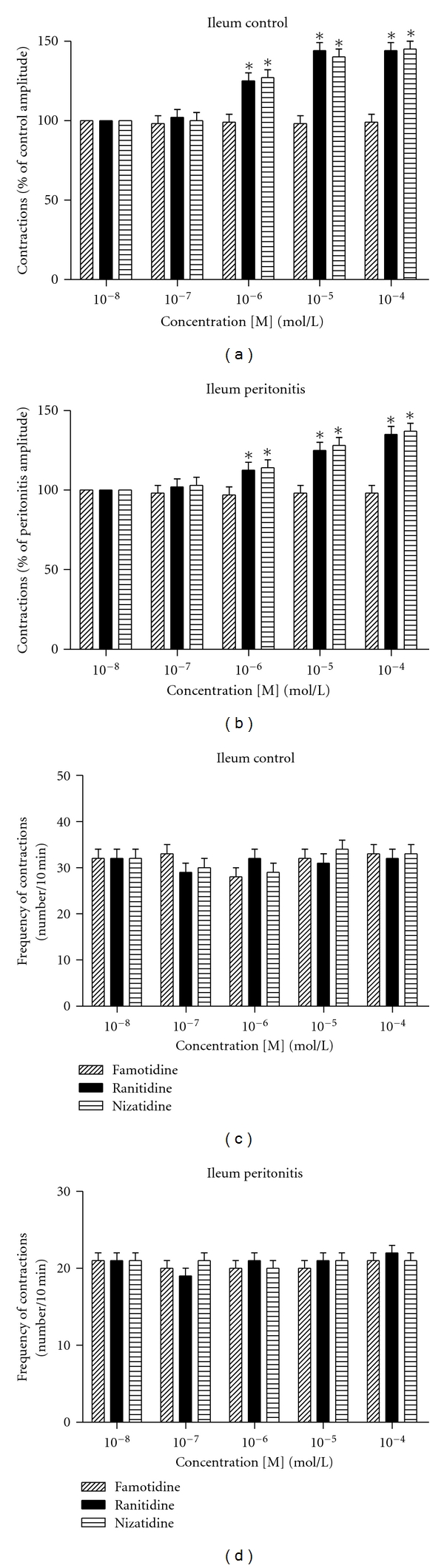
Amplitudes of the contractions induced by famotidine, ranitidine, and nizatidine. (a) Control group; (b) peritonitis group; both were calculated as the percentage of the initial contractions. (*P < 0.05 versus initial contractions; analysis of variance followed by Tukey test.) Changes induced by famotidine, ranitidine, and nizatidine in the frequency of spontaneous contractions. (c) Control group; (d) peritonitis group. Both were expressed as the number of contractions for 10 min.

The amplitude of spontaneous contractions of ileum muscle segments was studied after adding famotidine, ranitidine and nizatidine to the organ bath. Famotidine (10^−8^–10^−4 ^mol/L) caused no significant change on amplitude of spontaneous contractions in isolated ileum muscle segments, in both control and peritonitis groups. On the other hand, ranitidine (10^−8^–10^−4 ^mol/L) and nizatidine (10^−8^–10^−4 ^mol/L) significantly increased the amplitude of spontaneous contractions starting from 10^−6 ^mol/L in isolated ileum muscle segments, in both control and peritonitis groups, in a concentration-dependent manner. The increase in amplitude was higher in control group when compared to peritonitis group (Figures 3(a) and 3(b); *P* < 0.05).

The frequency of spontaneous contractions of ileum muscle segments was studied after adding famotidine, ranitidine, and nizatidine to the organ bath. Famotidine (10^−8^–10^−4 ^mol/L), ranitidine (10^−8^–10^−4 ^mol/L), and nizatidine (10^−8^–10^−4 ^mol/L) caused no significant change on frequency of spontaneous contractions in isolated ileum muscle segments in both control and peritonitis groups. There was also no significant difference between famotidine, ranitidine, and nizatidine in terms of effecting frequency of ileum muscle segments (Figures 3(c) and 3(d), *P* > 0.05).

## 4. Discussion

The first finding of our study is that peritonitis altered the spontaneous activity of the rat ileum by decreasing both the amplitude and the frequencies of the contractions in accordance with the previous studies reported recently by Koyluoglu et al. and Aydın et al. [12, 13]. The main findings are that PPIs, omeprazole, pantoprazole, and lansoprazole decreased amplitude and frequency of rhythmic contractions in both control and peritonitis groups. Therefore, H_2_ receptor antagonists, famotidine, ranitidine, and nizatidine increased motility in the ileum smooth muscle while they have no effect on frequency of ileum motility.

 Abdominal sepsis or peritonitis is also a major cause of morbidity and mortality in surgical intensive care units. Gastrointestinal dysmotility commonly accompanies peritonitis, and those patients suffering peritonitis are also exposed to the additive effects of sedatives or anesthetics in surgical intensive care units [13].

 PPIs and H_2_ receptor antagonists are the most commonly used drugs in acid-related diseases, for example, peptic ulcer, gastroesophageal reflux diseases (GERD), and Zollinger-Ellison syndrome. PPIs have been extensively studied for both efficacy and safety [11]. Of note, in several studies evaluating short-term treatment with PPIs, the investigators reported that it caused a delay in gastric emptying of solid meals in healthy subjects [14, 15]. However, the effects of PPIs on small bowel motility or transit are unclear; it is known that PPIs produce a dose-dependent delay in gastric emptying [10]. Investigators have previously shown that one month of PPI therapy was associated with reduced gallbladder motility [16]. This may show that PPIs reduce gallbladder motility and cause gallstones. Our results seem consistent with these previous studies. In our study we found that omeprazole, pantoprazole, and lansoprazole significantly decreased the amplitude and frequency of spontaneous contractions in both control and peritonitis groups. In both groups, the inhibitor effect of pantoprazole on ileum motility was significantly higher than omeprazole and lansoparazole. In peritonitis group, motility had been decreased by the peritonitis conditions itself. So, administration of PPIs in peritonitis group worsened the motility.

H_2_ receptor antagonists, in addition to their well-known gastric acid inhibitory effect, have prokinetic properties as well, thus stimulating gastrointestinal contractions and accelerating gastric emptying at gastric antisecretory doses [17, 18]. In fact, it has been suggested that some H_2_-receptor antagonists are more effective than several prokinetics in improving dyspeptic symptoms, gastric emptying and distention [19]. The prokinetic activity of the above agents derives mainly from their anticholinesterase activity. It is well known that in, intestinal smooth muscle, acetylcholine and its related stimulants produce contraction by activating muscarinic receptors (M_2_ and M_3_) [20]. In the study conducted by Unno et al., it has been suggested that the order of agonist efficacy for depolarization is the same as for Ca^2+^ store release that represents M_3_ activation. This finding suggests that M_3_ activation may contribute to voltage-dependent Ca^2+^ entry into the cell by potentiating the M_2_-mediated cationic current through both the indirect (Ca^2+^ store release) and direct pathways and so in turn by increasing the size of depolarization and the frequency of spike discharges. The idea is supported by our previous observation that depletion of Ca^2+^ stores attenuated carbachol-evoked depolarizations in single ileal muscle cells [21].

Indeed, *in vivo* [22] and *in vitro *studies [23] have shown that most H_2_-receptor antagonists exhibit weak or strong anticholinesterase activity, ranitidine and nizatidine being the more potent among the H_2_-receptor antagonists with respect to acetylcholinesterase inhibition [24]. The amplitude responses obtained with ranitidine and nizatidine were consistent with these data. In our study, in contrast to other H_2_ receptor antagonists, famotidine had no effect on amplitude in ileum smooth muscles. This may be related to different antimuscarinic effects profile of these H_2_ receptor antagonists.

Maher et al. [25] revealed that ranitidine shows its effects by promoting amplitude responses and does not have any effect on frequency responses. This finding is consistent with our results related to frequency. Any of H_2_ receptor antagonists we used had no effect on frequency responses in ileum smooth muscle.

In conclusion, PPIs and H_2_ receptor antagonists have contrary effects on ileum motility in both normal and peritonitis situations. The effects of these agents were parallel in control and peritonitis groups. The clinical implications of these findings need to be tested in surgical intensive care units, which might help in choosing the most appropriate drug for preventing the acid-related complications of patients with peritonitis.

## Figures and Tables

**Table 1:** Effects of proton pump inhibitors and H_2_ receptor antagonist agents on amplitude and frequency of the spontaneous contractions.

	Amplitude	Frequency
Omeprazole	Decreased	Decreased
Pantoprazole	Decreased	Decreased
Lansoprazole	Decreased	Decreased
Famotidine	No significant change	No significant change
Ranitidine	Increased	No significant change
Nizatidine	Increased	No significant change
